# Model-Based Meta-analysis of Rifampicin Exposure and Mortality in Indonesian Tuberculous Meningitis Trials

**DOI:** 10.1093/cid/ciz1071

**Published:** 2019-10-30

**Authors:** Elin M Svensson, Sofiati Dian, Lindsey Te Brake, Ahmad Rizal Ganiem, Vycke Yunivita, Arjan van Laarhoven, Reinout Van Crevel, Rovina Ruslami, Rob E Aarnoutse

**Affiliations:** 1 Department of Pharmacy, Radboud Institute of Health Sciences, Radboud University Medical Center, Nijmegen, The Netherlands; 2 Department of Pharmaceutical Biosciences, Uppsala University, Uppsala, Sweden; 3 Department of Neurology, Universitas Padjadjaran/Hasan Sadikin Hospital, Bandung, Indonesia; 4 Infectious Disease Research Center, Faculty of Medicine, Universitas Padjadjaran, Bandung, Indonesia; 5 Department of Biomedical Science, Pharmacology and Therapy Division, Universitas Padjadjaran/Hasan Sadikin Hospital, Bandung, Indonesia; 6 Department of Internal Medicine, Radboud Institute of Health Sciences, Radboud University Medical Center, Nijmegen, The Netherlands

**Keywords:** tuberculous meningitis, rifampicin, exposure response, optimal dosing, pharmacometrics

## Abstract

**Background:**

Intensified antimicrobial treatment with higher rifampicin doses may improve outcome of tuberculous meningitis, but the desirable exposure and necessary dose are unknown. Our objective was to characterize the relationship between rifampicin exposures and mortality in order to identify optimal dosing for tuberculous meningitis.

**Methods:**

An individual patient meta-analysis was performed on data from 3 Indonesian randomized controlled phase 2 trials comparing oral rifampicin 450 mg (~10 mg/kg) to intensified regimens including 750–1350 mg orally, or a 600-mg intravenous infusion. Pharmacokinetic data from plasma and cerebrospinal fluid (CSF) were analyzed with nonlinear mixed-effects modeling. Six-month survival was described with parametric time-to-event models.

**Results:**

Pharmacokinetic analyses included 133 individuals (1150 concentration measurements, 170 from CSF). The final model featured 2 disposition compartments, saturable clearance, and autoinduction. Rifampicin CSF concentrations were described by a partition coefficient (5.5%; 95% confidence interval [CI], 4.5%–6.4%) and half-life for distribution plasma to CSF (2.1 hours; 95% CI, 1.3–2.9 hours). Higher CSF protein concentration increased the partition coefficient. Survival of 148 individuals (58 died, 15 dropouts) was well described by an exponentially declining hazard, with lower age, higher baseline Glasgow Coma Scale score, and higher individual rifampicin plasma exposure reducing the hazard. Simulations predicted an increase in 6-month survival from approximately 50% to approximately 70% upon increasing the oral rifampicin dose from 10 to 30 mg/kg, and predicted that even higher doses would further improve survival.

**Conclusions:**

Higher rifampicin exposure substantially decreased the risk of death, and the maximal effect was not reached within the studied range. We suggest a rifampicin dose of at least 30 mg/kg to be investigated in phase 3 clinical trials.

## Teaser

Through an individual participant meta-analysis, we characterized the relationship between rifampicin exposure and tuberculous meningitis mortality. Higher rifampicin exposure substantially decreased the risk of death, and doses of at least 30 mg/kg should be moved forward to phase 3 clinical trials.

Tuberculous meningitis (TBM) affects 100 000 individuals worldwide annually and has been called the worst possible form of tuberculosis (TB) [1, 2]. Outcomes are generally poor with mortality of >30% and frequent neurological sequelae [3–6]. The antimicrobial treatment of TBM is traditionally based on the guidelines for pulmonary TB, a combination therapy with rifampicin as the pivotal component [7]. The crucial role of rifampicin is underlined by the excessively high mortality rates in patients with TBM with resistance to rifampicin [8, 9]; this, even though it is known that penetration of rifampicin into cerebrospinal fluid (CSF) is very limited [10].

The standard dose of rifampicin (10 mg/kg) was selected decades ago, partly based on cost considerations not relevant today [11]. A growing body of preclinical and clinical evidence suggests that higher rifampicin doses and subsequent increased drug exposures accelerate the rate of mycobacterial clearance and thereby improve outcomes in pulmonary TB [12–19]. A series of phase 2 clinical trials investigating intensified regimens for TBM using high-dose rifampicin has been conducted in Bandung, Indonesia [20–22]. Given the modest number of patients and the restricted range of rifampicin doses within each study, the separate datasets provided limited possibilities for exposure-response analysis.

In this work we conducted a joint analysis using advanced modeling methodology, pooling the data from all 3 trials. For the mortality data, we conducted a time-to-event analysis using parametric hazard models, which yield higher statistical power compared to the conventionally used Cox regression [23] and allow for easy testing of nonlinear covariate relationships, including the often used Hill function (E_max_) function for concentration-effect relationships. Our objective was to characterize the population pharmacokinetics of high-dose rifampicin in plasma and CSF and to evaluate the relationship between individual exposures and mortality. In addition, we performed an exposure–safety analysis relating individual rifampicin levels to occurrence of adverse events. Together this will help to identify the appropriate rifampicin dose to attain desirable exposures, improving the survival of patients with TBM.

## METHODS

### Studies and Data

Data originated from 3 randomized phase 2 trials comparing oral rifampicin 450 mg (approximately 10 mg/kg in this patient population) to intensified 14- or 30-day regimens including 750 mg (17 mg/kg), 900 mg (20 mg/kg), or 1350 mg (30 mg/kg) orally, or a 600-mg (13 mg/kg) intravenous infusion (1.5 hours), next to other first-line TB drugs and adjunctive dexamethasone. All 3 studies were conducted in Bandung, Indonesia, and included adult patients with definite (microbiologically proven), probable, or possible TBM. Details on study design and procedures can be found in the Supplementary Materials and the original publications [20–22]. In brief, rich pharmacokinetic sampling (6 time-points) was performed at day 2 ± 1, and for 2 of the studies also at day 12 ± 4, both during the critical initial phase of treatment of TBM. Single CSF samples were collected at 3–9 hours after dose. The rifampicin plasma and CSF concentrations were quantified with validated high-performance liquid chromatography or ultra-performance liquid chromatography methods [20, 21]. The neurological status of the patients was graded with the Glasgow Coma Scale (GCS) score and the patients were followed for 6 months recording survival. Adverse events were recorded and graded based on the Common Terminology Criteria for Adverse Events.

### Software

Data management, plotting, and postprocessing of results were performed in R (R Foundation for Statistical Computing, Vienna, Austria), partially using the Xpose package (Department of Pharmaceutical Biosciences, Uppsala University, Uppsala, Sweden). The modeling and simulations were performed in NONMEM 7.4 (Icon Development Solutions, Ellicott City, Maryland), aided by PsN (Department of Pharmaceutical Biosciences, Uppsala University, Uppsala, Sweden) and Pirana (Certara, Princeton) [24].

### Modeling

Nonlinear mixed-effects methodology was utilized to describe the pharmacokinetic data. Previously published rifampicin population models were used as a starting point and further refined on the current data [25, 26]. Potential predictors interindividual variability in exposure were evaluated in a stepwise manner using log-likelihood ratio testing with a significance level of 0.05. Parametric time-to-event models were used to characterize the survival. The following functions were evaluated: constant, Weibull, Gompertz, and exponentially declining hazard. Impact of patient characteristics and different individual rifampicin exposure metrics (obtained from the pharmacokinetic model) were tested on the hazard function. The selected relation with rifampicin exposure was further challenged in a nonparametric bootstrap procedure (500 samplings). The relation between individual rifampicin exposures and risk of developing adverse events was investigated with logistic regression models. The models were evaluated according to established best practice using, among other things, visual predictive checks (VPCs) and parameter precision [27]. Last, the rifampicin exposures predicted to give a desired effect were translated to recommended doses. Details on the population (10 000 virtual patients) forming the basis for the evaluation are included in the Supplementary Materials.

## RESULTS

### Patients and Analyses

A total of 148 patients from the 3 studies were available for the joint analysis. The patients were relatively young, 55% were male, and 95% had grade 2 or 3 TBM. Further demographic information is summarized in Table 1. The pharmacokinetic analyses included 133 of those individuals (15 of the total 148 were excluded as no pharmacokinetic observations were available) with 1150 observed rifampicin concentrations (including 170 from CSF).

**Table 1.** Patient Characteristics in the Total Patient Population (N = 148)

Characteristic	Median (Range) or No. (%)
Sex	
Male	81 (55%)
Female	67 (45%)
Age, y	30 (16–81)
Weight, kg	46 (34–78)
HIV infection	18 (12%)
Diagnosis, definite TBM	83 (56%)
Baseline GCS score	13 (3–15)
CSF protein, mg/dL	165 (9–3869)
CSF neutrophils, cells/µL	24 (0–874)
CSF leukocytes, cells/µL	122 (0–1397)
CSF/blood glucose ratio	0.24 (0.03–1)

Abbreviations: CSF, cerebrospinal fluid; GCS, Glasgow Coma Scale; HIV, human immunodeficiency virus; TBM, tuberculous meningitis.

### Pharmacokinetic Model

Based on our pharmacokinetic model, the oral bioavailability of rifampicin was estimated at 77.6% (95% confidence interval [CI], 70.9%–84.3%). Rifampicin clearance was found to decrease with increasing plasma concentrations following Michaelis Menten kinetics and the intrinsic clearance was 47.9% (95% CI, 27.5%–68.3%) higher after day 4 due to the phenomenon of autoinduction. Volume of distribution was estimated to be 19.3% (95% CI, 3.6%–26.4%) lower at the later sampling occasion compared to the first. Concentrations in CSF were directly related to the plasma concentrations through the means of a penetration coefficient (5.5%; 95% CI, 4.5%–6.4%) and a half-life for distribution (2.1 hours; 95% CI, 1.3–2.9 hours). A correlation between the penetration coefficient and individual protein concentrations in CSF was identified. The relation was described through a linear function with log-transformed protein concentration leading to a 63% (95% CI, 57%–120%) increase in the penetration coefficient with each 10-fold change in protein levels. A full description of the final pharmacokinetic model is available in the Supplementary Materials, including Supplementary Table 1 with parameter values, Supplementary Figure 1 showing VPCs of plasma concentrations per dose and occasion, and Supplementary Figure 2 showing a prediction-corrected VPC of CSF concentrations, as well as the NONMEM code for the model. The model was utilized to generate individual exposure metrics to evaluate in the survival model: area under the rifampicin plasma concentration curve from 0 to 24 hours after dose (AUC_0-24h_) and peak concentration (C_max_) in plasma and CSF at day 2 ± 1. Typical exposures were imputed based on the model, patient characteristics, and the given dose for the 15 patients missing observed pharmacokinetic data. A histogram depicting the individual exposures and median per dose level can be seen in Supplementary Figure 3.

### Survival Model

**Figure 1.**
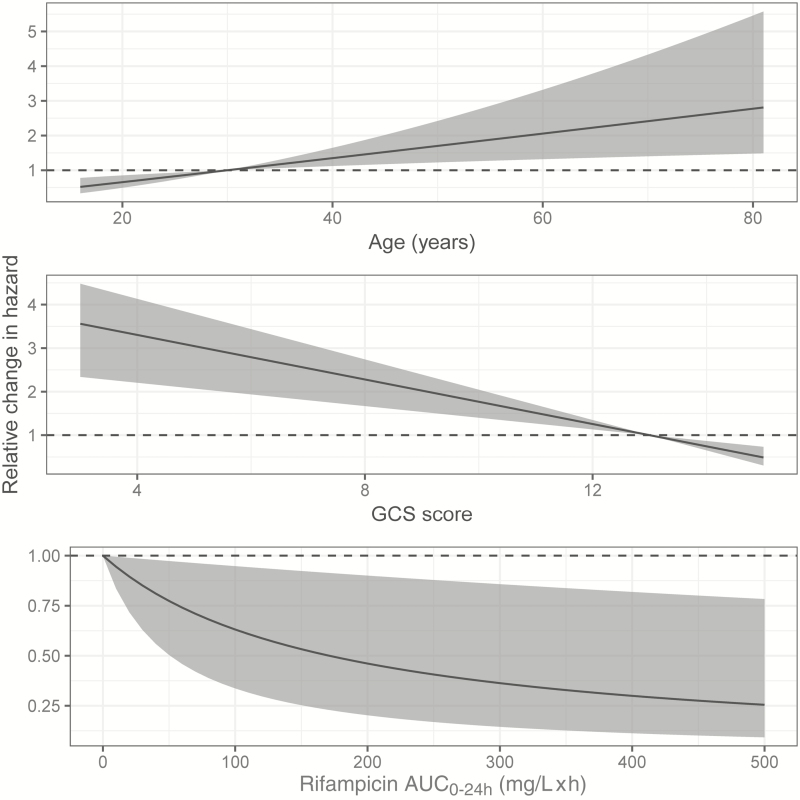
Visualization of the impact of predictors on the hazard governing survival. Depicted ranges of age, Glasgow Coma Scale (GCS) score, and plasma rifampicin area under the rifampicin plasma concentration curve from 0 to 24 hours after dose (AUC_0-24h_) at day 2 ± 1 correspond to the observed values in the population included in this analysis. The gray shaded areas represent 90% confidence intervals based on bootstrap results.

**Figure 2.**
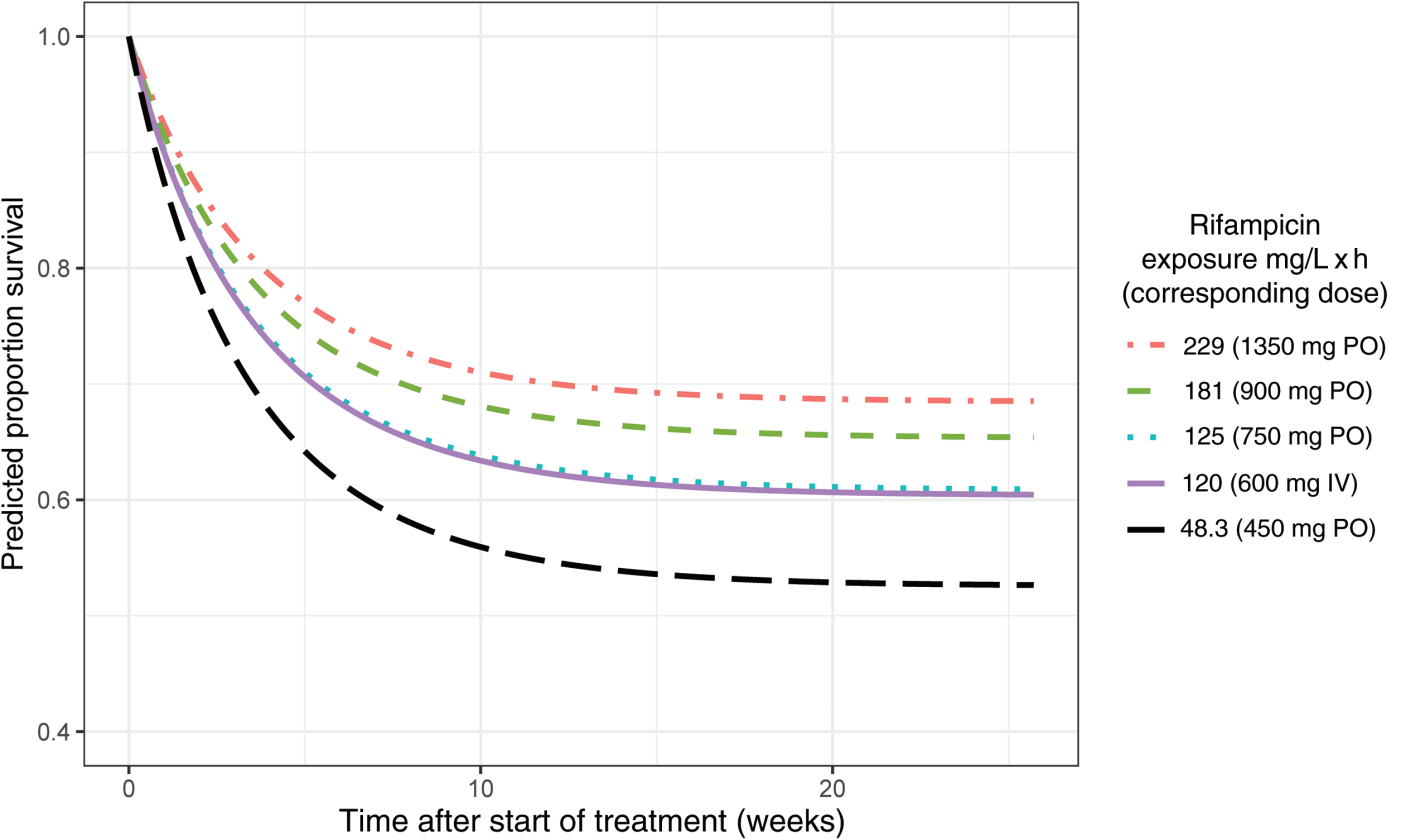
Model-predicted proportion survival over time representative for typical patients (age 30 years, baseline Glasgow Coma Scale score = 13) per plasma rifampicin exposure level (area under the rifampicin plasma concentration curve from 0 to 24 hours after dose at day 2 ± 1). Abbreviations: IV, intravenously; PO, orally.

An exponentially declining base hazard model described the survival data the best; Supplementary Table 2 lists a comparison of the likelihood for the various evaluated models. In univariate analysis, age (*P* = .009) and baseline GCS score (*P* = .0003) were found to significantly affect the hazard, whereas sex, human immunodeficiency virus (HIV) infection, body weight, and CSF neutrophil or protein concentrations did not (all *P* ≥ .05). Out of the individual exposure metrics, rifampicin plasma AUC_0-24h_ was a better predictor for survival than plasma C_max_ or CSF AUC_0-24h_. An E_max_ equation allowing for 100% decrease in the hazard was selected to describe the relationship as no maximal effect could be characterized. The plasma AUC_0-24h_ at day 2 ± 1 corresponding to a 50% decrease in the hazard was estimated to 171 mg/L × hour (relative standard error [RSE], 86%). The bootstrap evaluation confirmed the magnitude but also the relatively large uncertainty in the estimate of this parameter (median, 203 mg/L × hour; 90% CI, 51–1804). Further details on the evaluated exposure-response relationships are included in Supplementary Table 3 and the shape of the included covariate relations is displayed in Figure 1. All parameter estimates of the final model are included in Table 2, and Supplementary Figure 4 demonstrates the good fit of the model through Kaplan-Meier VPCs per dose group. The predicted survival over time per dose group is shown in Figure 2, demonstrating that an increase from 450 mg to 1350 mg could be expected to increase survival from approximately 50% to 70% in a similar population.

**Table 2.** Parameters for Final Parametric Survival Model Including Predictive Relations and Estimates of Uncertainty

Parameter	Value	RSE
BASE^a^, base hazard, day ^−1^	0.0286	41%
k^a^, rate constant exponential decline in hazard, day ^−1^	0.0333	17%
θ _GCS_^a^, GCS effect	−0.256	28%
θ _age_^a^, age effect	1.04	39%
θ _RIF_^a^, rifampicin EC_50_, mg/L × h	171	86%

Abbreviations: BASE, base hazard; EC_50_, exposure giving half of maximal effect; GCS, individual Glasgow Coma Scale score at baseline; RSE, relative standard error.

^a^Parametric hazard model: h(t) = BASE × exp(−k × t) × (1 + θ _GCS_ × [GCS-13]) × (age/30)^θ_age_ × (1 – AUC_RIF_ / [θ _RIF_ + AUC_RIF_]), where AUC_RIF_ is the estimated individual 24-hour area under the concentration curve for rifampicin day 2 ±1 on study.

### Safety Model

The risk of occurrence of at least 1 adverse event for each patient was 65% (RSE, 8%) in the first 2 studies and 83% (RSE, 6%) in the last study. The risk of occurrence of at least 1 serious adverse event (grade 3 and 4) was 23% (RSE, 15%) and not scientifically different between studies. There was no significant effect of rifampicin exposure on either of these risks (*P* = .47 and *P* = 1, respectively).

### Predicted Optimal Dose

Different doses between 450 mg (~10 mg/kg) and 1800 mg (~40 mg/kg) were evaluated for probability of attaining early rifampicin exposures higher than 171 mg/L × hour (estimated to give 50% of maximal effect) or 300 mg/L × hour (an exposure within the observed range generating about 65% of maximal effect), as summarized in Table 3. A dose of 1800 mg would be needed to achieve the higher target in 95% of patients with TBM.

**Table 3.** Evaluation of Probability of Target Attainment for Different Rifampicin Doses

Rifampicin Dose, mg	Approximate Dose/Body Weight, mg/kg	Probability of Plasma AUC_0-24h_ at Day 2 ± 1 > 171 mg/L × h, %	Probability of Plasma AUC_0-24h_ at Day 2 ± 1 > 300 mg/L × h, %
450	10	0.47	0
900	20	67.8	11.4
1350	30	97.6	70.1
1800	40	99.8	94.5

Abbreviation: AUC_0-24h_, area under the rifampicin plasma concentration curve from 0 to 24 hours after dose.

## DISCUSSION

In this individual patient meta-analysis including data from 3 phase 2 TBM trials investigating intensified rifampicin treatment, we found 3 main factors affecting the chance of survival up to 6 months after start of treatment: disease severity at start of study (quantified by GCS score), age, and rifampicin plasma exposure level. An increase in dose from 450 mg (10 mg/kg) to 1350 mg (30 mg/kg) orally is predicted to increase survival at 6 months from 50% to 70%, and higher doses would further reduce mortality. Based on our analysis an oral dose of 1800 mg is likely to attain desired exposures in the vast majority of patients.

Previous analyses of one of the trials included in this work also found improved survival with higher rifampicin dose or exposure [20, 26, 28], whereas Heemskerk et al detected no significant effect when 15 mg/kg was compared to the standard 10 mg/kg dose [5]. Given the shape of the exposure–response relationship that we could characterize in this analysis (Figure 1), we suggest that this lack of significant improvement is due to the rather limited impact on survival expected with such a modest dose increase (8% for a dose of 750 mg compared to 450 mg). Our results suggest that the higher the rifampicin exposure the better the survival, and that the exposure predicted to give 50% of the maximal effect is higher than previously suggested targets [26, 28]. Doses of at least 1350 mg (approximately 30 mg/kg) are needed to give 50% of the maximal effect with high probability, while 1800 mg (approximately 40 mg/kg) would be the recommended dose when setting a more ambitious target (Table 3). We found no relation between individual rifampicin exposures and adverse events, and results from studies in pulmonary TB demonstrate that 35–40 mg/kg rifampicin can be administered safely [16, 17]. This supports the doses selected in the ongoing RifT study investigating up to 35 mg/kg rifampicin [29]. It should be noted that the precise shape of the relationship between rifampicin exposure and survival remains uncertain given the relatively low parameter precision obtained.

Our analysis is in line with previous analyses that have shown that more severe disease and old age increase mortality [30–34]. HIV infection is also known to be associated with increased TBM mortality [3, 4], but did not affect outcomes in this analysis, probably due to the small number of HIV-infected patients in our dataset (12%).

The pharmacokinetic analysis showed that only a small proportion of rifampicin penetrated to the CSF (around 5%), that CSF concentrations were directly linked to plasma concentrations, and that distribution to CSF did not saturate within the studied range of rifampicin exposures. The interindividual variability in the penetration was 36% (coefficient of variation, RSE 14%). We found that higher levels of proteins in the CSF were linked to higher rifampicin concentrations. This is probably not a causal relationship, but may reflect more leaky barriers that let both proteins and rifampicin through. In healthy individuals CSF is nearly protein-free [35]; consequently, drugs in CSF are largely considered protein-unbound [36]. This dogma could be questioned in meningitis patients where protein levels in CSF can be substantially increased [4]. In plasma, rifampicin is around 80% protein-bound [37, 38]. Albumin accounts for 30%–40% of the binding in serum, which leaves a possible important role for γ-globulin binding proteins [37, 39]. In the analyzed studies, only total CSF protein was measured, but in a partly overlapping subset of 29 patients from the same cohort, we measured serum and CSF albumin batchwise (Supplementary Figure 5). Albumin constituted a median of 51% (range, 26%–95%) of total CSF protein. This information is not sufficient to draw conclusions regarding rifampicin binding in CSF and it would be informative to directly measure the free fraction of rifampicin in CSF in future trials.

Another common hypothesis in meningitis treatment is that drug concentrations in CSF should be more closely linked to outcome than plasma levels since the target site is in the brain and CSF is closer to the brain than plasma. This was not supported in our analysis where plasma exposure was found to be a better predictor of survival than CSF exposure. This could be due to different reasons, for example, that the actual individual CSF exposure was not well estimated based on single point concentration measurements or that CSF concentrations are not always a good marker for brain exposure [40–42].

Our analysis has a number of limitations to be considered. First, data were combined from 3 separate studies with slightly different inclusion and exclusion criteria. The length of the intensified treatment varied between the studies (14 or 30 days); this was evaluated as a covariate in the survival model but not found to be significant. Furthermore, not all patients had confirmed TBM (56%) and this proportion varied between studies. All 3 studies were conducted in Indonesia, potentially limiting the global applicability of the results. Included patients were allowed to have had up to 3 days of rifampicin-containing therapy before the start of the study. The exact number of prestudy treatment days was not recorded and could therefore not be adjusted for. This also mandated a somewhat simplistic way of modeling the autoinduction of rifampicin clearance with a stepwise change over time rather than a more plausible gradual change as earlier described [25, 43]. Total rifampicin concentrations were measured and modeled while only unbound concentrations are generally considered to exhibit pharmacological effects. However, it has recently been shown that the free fraction remains constant over a wide range of rifampicin concentrations [38], mitigating the risk of bias when evaluating total concentrations as done here. The exposure-safety analysis performed is rather simplistic, evaluating all type of adverse events jointly. A more in-depth analysis focusing specifically on hepatoxicity might bring additional insights. Last, no maximum to the rifampicin effect on survival could be estimated, most likely due to the limited range of exposures included in the analysis and statistical power. An implication is that no target exposure can be derived based on this model and that predictions of survival for exposures substantially above the range here studied would be highly uncertain.

In conclusion, by combining clinical trial data and employing a model-based analysis, we can support that higher doses of rifampicin improve survival in persons with TBM. Besides intensified treatment using high doses of rifampicin, other aspects of care are relevant for outcome of TBM treatment, including timely diagnosis and treatment initiation, better supportive care, and possibly, host-directed therapy. Still, increasing the dose of rifampicin may be one of the simplest ways to improve treatment outcomes. Hopefully, definitive evidence for such an intervention will come from a planned double-blind randomized controlled clinical trial that will evaluate rifampicin doses of 1500–1800 mg in 500 patients in Uganda, South Africa, and Indonesia (ISRCTN registry number 15668392). The meta-analysis presented herein was part of the work paving the road for this unique phase 3 trial.

## Supplementary Data

Supplementary materials are available at *Clinical Infectious Diseases* online. Consisting of data provided by the authors to benefit the reader, the posted materials are not copyedited and are the sole responsibility of the authors, so questions or comments should be addressed to the corresponding author.

ciz1071_suppl_Supplementary_Material

## Notes

***Acknowledgments.*** The authors thank all of the patients and the staff who participated in the conduct of the original clinical studies, without whom this analysis would not have been possible. The authors are also grateful for the technical support with computational facilities that was provided by Dr Arthur Pistorius, Radboud University Medical Center.

***Potential conflicts of interest.*** The authors: No reported conflicts of interest. All authors have submitted the ICMJE Form for Disclosure of Potential Conflicts of Interest.
