# Supplementary material for: Model-Based Meta-analysis of Rifampicin Exposure and Mortality in Indonesian Tuberculous Meningitis Trials
Source: Clin Infect Dis. 2019 Oct 30;71(8):1817–23. doi: 10.1093/cid/ciz1071 (PMC7643733; doi:10.1093/cid/ciz1071)
Supplement: ciz1071_suppl_Supplementary_Material [file ciz1071_suppl_supplementary_material.pdf]

Online Data Supplement

Model-based meta-analysis of rifampin exposure and mortality in Indonesian tuberculosis meningitis trials

Elin M Svensson[1,2], Sofiati Dian[3,4], Lindsey te Brake[1], Ahmad Rizal Ganiem[3,4], Vycke Yunivita[4,5], Arjan van Laarhoven[6], Reinout van Crevel[6], Rovina Ruslami[4,5], Rob E Aarnoutse[1]

1. Department of Pharmacy, Radboud Institute of Health Sciences, Radboudumc, Nijmegen, The Netherlands
2. Department of Pharmaceutical Biosciences, Uppsala University, Uppsala, Sweden
3. Department of Neurology, Universitas Padjadjaran/Hasan Sadikin Hospital, Bandung, Indonesia
4. Infectious Disease Research Center, Faculty of Medicine, Universitas Padjadjaran, Bandung, Indonesia
5. Department of Biomedical Science, Pharmacology and Therapy Division, Universitas Padjadjaran/Hasan Sadikin Hospital, Bandung, Indonesia
6. Department of Internal Medicine, Radboud Institute of Health Sciences, Radboudumc, Nijmegen, The Netherlands

Table of Content

Study designs and patients 3

Bioanalysis 4

Pharmacokinetic model 5

Table E1. Parameter estimates including precision of final pharmacokinetic plasma and CSF model 6

Figure E1. Visual predictive check of plasma PK model 7

Figure E2. Visual predictive check of CSF PK model 8

Figure E3. Histogram of individual rifampin exposures 9

Population for dose evaluations 10

Survival model 11

Table E2. Fit of different evaluated base hazard models 11

Table E3. Evaluated relationships between rifampin exposure and base hazard model 11

Figure E4. Evaluation of survival model 12

Albumin concentrations 13

Figure E5. Albumin in CSF and serum 13

NONMEM code pharmacokinetic model 14

NONMEM code survival model 18

References 21

Study designs and patients

Study 1 by Ruslami *et al.* was an open-label, randomized, clinical trial with a factorial design (1). High-dose rifampicin and standard-dose or high-dose moxifloxacin were assessed as part of a four-drug regimen for tuberculous meningitis. Patients were first randomly assigned to receive a regimen with rifampicin at a standard dose (450 mg once daily, about 10 mg/kg, orally) or high dose (600 mg once daily, about 13 mg/kg, intravenously). The patients were then randomly assigned to receive oral ethambutol (750 mg), standard dose moxifloxacin (400 mg), or high-dose moxifloxacin(800 mg). All patients were given oral isoniazid (300 mg/day), pyrazinamide (1500 mg/day), and pyridoxine (50 mg/day). The intensified regimens were given for the first two weeks of treatment and then all patients were given standard treatment.

All patients older than 14 years with definite, probable, or possible tuberculous meningitis were eligible for the study. Exclusion criteria were failure to do a diagnostic lumbarpuncture, evidence of bacterial or cryptococcal meningitis, treatment for tuberculosis for more than seven days before admission, a history of tuberculous meningitis, pregnancy, lactation, a known contraindication to moxifloxacin, alanine aminotransferase activity more than five times the upper limit of normal, known hypersensitivity or intolerance to rifampicin or moxifloxacin, rapid clinical deterioration during the screening process, and absence of informed consent.

Pharmacokinetic sampling was done in the first 3 days of the study. Serial blood sampling was done just before and at 1, 2, 4, 6, and 24 h after dosing. Two samples of cerebrospinal fluid were taken on the same day as the blood samples and more than 24 h after the first sampling of cerebrospinal fluid, between 3 h and 6 h and 6 h and 9 h after drug administration, respectively.

Study 2 by Yunivita *et al.* was an explorative, open-label, randomized, three-arm, two-period pharmacokinetic and safety/tolerability study (2). Thirty subjects were randomized to three groups who received fixed daily doses of rifampin 750 mg (ca. 17 mg/kg) orally, 900 mg (ca. 20 mg/kg) orally or 600 mg (ca. 13 mg/kg) i.v. for 14 days. After 14 days, the subjects were treated with a standard TB regimen according to the Indonesian National TB programme.

Adult patients (age ≥17 years) with definite, probable or possible TBM, as determined by a scoring system [10], were eligible for inclusion in the study. Subjects were excluded if they had been treated for TB for ≥3 days before admission, had alanine aminotransferase (ALT) more than five times the upper limit of normal, had a positive urine pregnancy test, had a history of hypersensitivity to rifampicin, failure to do a diagnostic lumbar puncture, or evidence of bacterial or cryptococcal meningitis.

The first sampling for pharmacokinetic assessment was done within the first 3 days of drug administration and a second sampling took place ≥9 days after treatment initiation. Serial venous blood samples were collected just before and at 1, 2, 4, 8 and 12 h after administration of the oral dose or the start of i.v. administration. A CSF sample was taken on the same days as the blood samples, between 3 h and 6 h after drug administration. Subjects had an overnight fast from 23:00 h on the day before until 2 h after administration of the study drug.

Study 3 by Dian *et al.* was a double-blinded, randomized, placebo-controlled trial with three parallel arms (3). Patients were assigned a standard (450 mg, one active and two placebo tablets), double (900 mg, two active and one placebo tablet), or triple (1350 mg, three active tablets) dose of

rifampin for 30 days, in addition to other TB drugs, according to Indonesian national guidelines. Randomization occurred in variable block sizes and was stratified by BMRC grade.

The inclusion criteria were: 15 years of age or above, providing informed consent (ev. through representative) for the study and storage of samples, with clinical suspicion of TBM and CSF/blood glucose ratio < 0.5. None or less than 3 days of anti-tuberculosis chemotherapy taken for the current infection. Females who are able to become pregnant had to agree to use at least one effective form of nonhormonal contraception. Patients were excluded if no CSF data were available to enable a diagnosis of microbiologically confirmed (definite) or probable TBM, had liver or kidney dysfunction, were pregnant or breastfeeding, had confirmed Cryptococcus meningitis in HIV-positive patients; or diagnosed as bacterial meningitis based on clinical assessment and routine CSF examination. Rapid clinical deterioration at time of presentation and history of hypersensitivity/intolerance to rifampin were also exclusion criteria.

Pharmacokinetic sampling was performed twice, at day 2±1 and then at day 10±1 of study treatment. On each sampling day, serial blood sampling was performed just before and at 1, 2, 4, 8, and 12 h after dosing. CSF samples were collected on both sampling days, between 3 and 9 h after dosing. Patients had an overnight fast before sampling and remained fasted until 2 h after the administration of the study drugs.

The adverse event recording in all three studies utilized the common terminology criteria for adverse events (CTCAE) system version 4. In study one and two the following categories were included: hepatotoxicity, nausea, vomiting, abdominal discomfort, diarrhea, rash, purpura and pruritus, plus cardiac events in study one only. In study three additional categories (anemia, leukopenia, thrombocytopenia) were included. For the purpose of our exposure-safety analysis only adverse events in the categories included in study one and two were considered.

Bioanalysis

In study 1 the plasma and CSF concentrations of rifampin were assessed at Radboud University Nijmegen Medical Centre, Nijmegen, Netherlands, using a validated high performance liquid chromatography assays that performed well in an international quality control programme (4). The accuracy for standard concentrations was between 99.8% and 100.4%. The intra-assay and inter-assay coefficients of variation were less than 4% for a concentration of 0.26–30 mg/L. The lower limit of quantification was 0.26 mg/L.

In study 2 and 3 analysis of rifampicin concentrations in plasma and CSF was performed at the Pharmacokinetic Laboratory of the Faculty of Medicine of Universitas Padjadjaran using a validated ultraperformance liquid chromatography (UPLC) method. Accuracy was between 95.1% and 102.4% for plasma samples and between 94.5% and 100.7% for CSF samples, depending on the concentration level. The intraday and interday coefficients of variation were <4.2% over the 0.26–30 mg/L concentration range for rifampicin in plasma and <3.4% over the 0.25–30 mg/L range in CSF.

Pharmacokinetic model

Two disposition compartments were needed to capture profiles from both oral and intra venous administration. The absorption of rifampin administered orally was described with a dynamic transit model with an estimated number of transit compartments (5). Rifampin clearance and first-pass metabolism were modeled through a well-stirred liver model with saturable intrinsic clearance (following Michaelis-Menten kinetics) as previously described by Chirehwa *et al* (6). Hepatic plasma flow and liver volume were fixed to 50 L/h and 1 L, respectively. Rifampin protein binding was assumed to be 20%. Oral bioavailability (pre hepatic first-pass metabolism) was estimated to almost 80%. A nonlinear relation with dose as earlier described in models without a liver compartment could not be detected here (7, 8), maybe since a type of nonlinearity already is included through saturation of the first-pass metabolism. Allometric scaling with fat-free mass as body size descriptor and coefficients fixed to the theoretical values (0.75 for clearance and 1 for volume) was included *a priori* based on experience form previous . The expected autoinduction was described with a factor change in intrinsic clearance from day 4 on study and onwards, and was estimated to an increase of close to 50%. A more mechanistically plausible gradual change in clearance intrinsic was not meaningful to evaluate given that the exact day of rifampin treatment was unknown (patients could be included in the studies until maximally 3 days after start of rifampin-containing treatment). Volume of distribution (central and peripheral) was estimated to be about one fifth lower at the later sampling occasion compared to the first. This could be due to the generally . Samples below the limit of quantification were excluded in the estimation but retained in the VPC plots to diagnose the model's ability to describe low concentrations. The final parameter estimates are summarized in Table E1 and Figure E1 depicts the VPCs per dose level and sample occasion.

The CSF concentrations were directly linked to the plasma concentrations via a half-life for distribution and a partition coefficient. The relation was coded without actual mass transfer in the model. A linear relation between log10 transformed levels of protein in CSF and partition coefficient was detected, increasing levels of protein resulting in a higher partition coefficient, potentially signifying a generally more leaky blood-brain barrier. The final parameter estimates are summarized in Table E1 and Figure E2 depicts the prediction corrected VPC (9).

Individual exposure metrics ($AUC_{0\text{-}24h}$ and Cmax per sampling occasion for both plasma and CSF) were derived from the final model. The commented NONMEM code is available last in this supplementary material document.

Table E1. Parameter estimates including precision of final pharmacokinetic plasma and CSF model

Parameter	Value (RSE)	IIV (RSE)	IOV (RSE)
**Plasma model**			
Intrinsic clearance [L/h]	40.5 (8.5%)		24.8% (11%)
Km [mg/L]	16.1 (21%)		
Volume of distribution – central [L]	7.38 (29%)	147% (17%)	
Intercompartmental clearance [L/h]	93.1 (25%)	126% (23%)	
Volume of distribution – peripheral [L]	26.2 (5.0%)		
Rate of absorption [h^{-1}]	1.41 (17%)	88.3% (20%)	
Mean transit time [h]	0.67 (12%)	67.7% (14%)	
Number of transit compartments	4.23 (14%)		
Bioavailability [%]	77.6 (4.4%)	134% (11%)	
Induction[a] [%]	47.9 (22%)		
Difference on volumes after day 4 [%]	-19.3 (19%)		
Additive residual error [mg/L]	0.1 (33%)		
Proportional residual error [%]	24.3 (2.8%)		
**CSF model**			
Half-life distribution [h]	2.07 (20%)		
Penetration coefficient [%]	5.46 (9.2%)	36.3% (14%)	
Effect of protein[b] [%]	63.1 (46%)		
Additive residual error [mg/L]	0.19 (9%)		

Abbreviations: RSE, relative standard error; IIV, inter-individual variability; IOV, inter-occasion variability

[a] Increase in intrinsic clearance, factor change after day 4

[b] Effect of one log10 increase in CSF protein levels on the penetration coefficient. Parameter estimation centered on log10 of median CSF protein levels (165 cells/mL).

Figure E1. Visual predictive check of plasma PK model

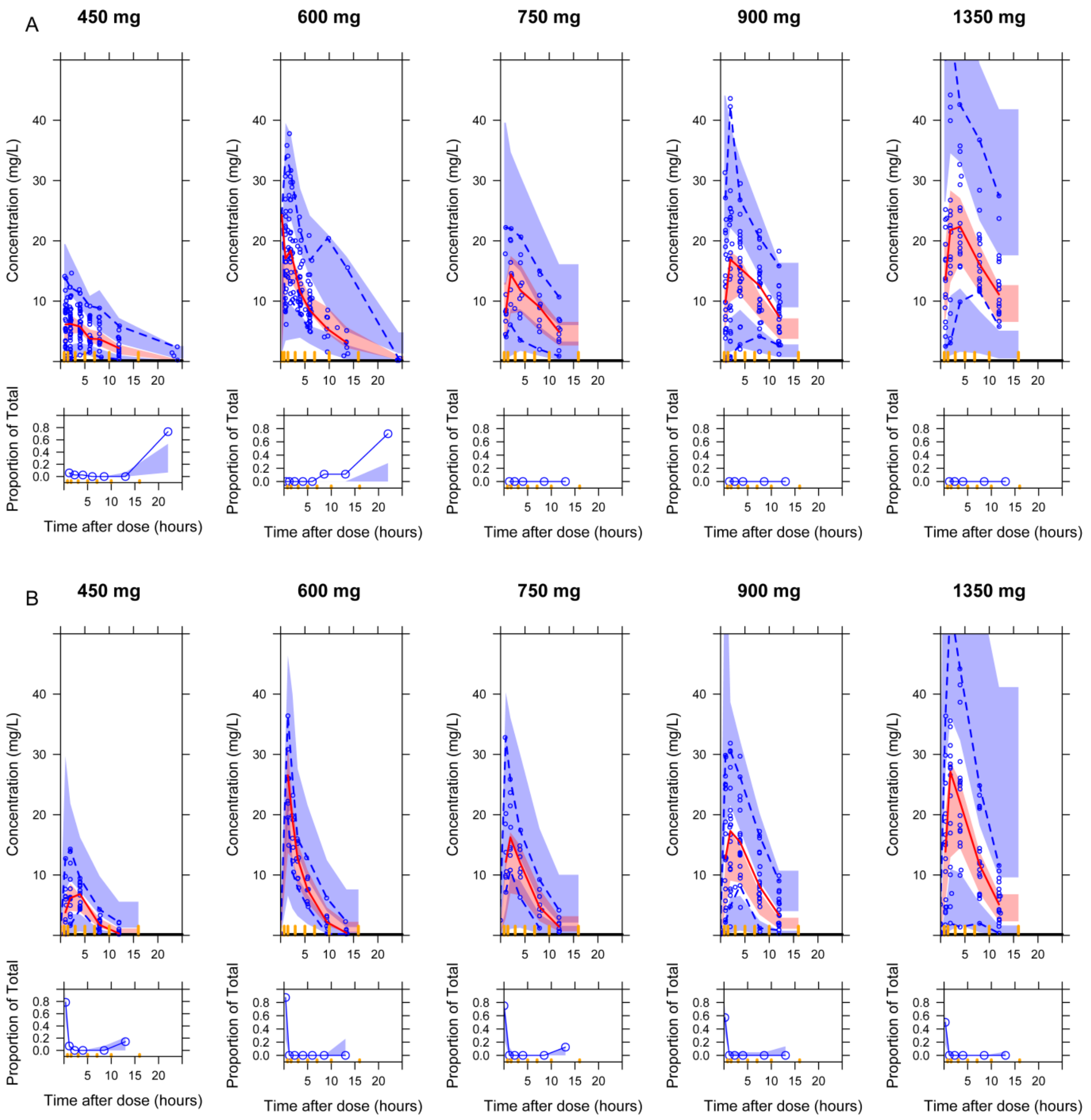

VPCs per dose and early (day 1-3, panel A) and later sampling (day 8-16, panel B). In the upper part of each panel the blue rings represent the observed rifampicin concentrations, the lines represent the 2.5th, 50th and 97.5th percentiles of the observed data, and the shaded areas are the 95% confidence intervals of the same percentiles based on data simulated by the final model. In the lower part of each panel the blue rings represent the observed proportions samples below the limit of quantification per bin (indicated by the yellow tick marks), and the shade area represents the 95% confidence intervals of the same proportions based on data simulated by the final model.

Figure E2. Visual predictive check of CSF PK model

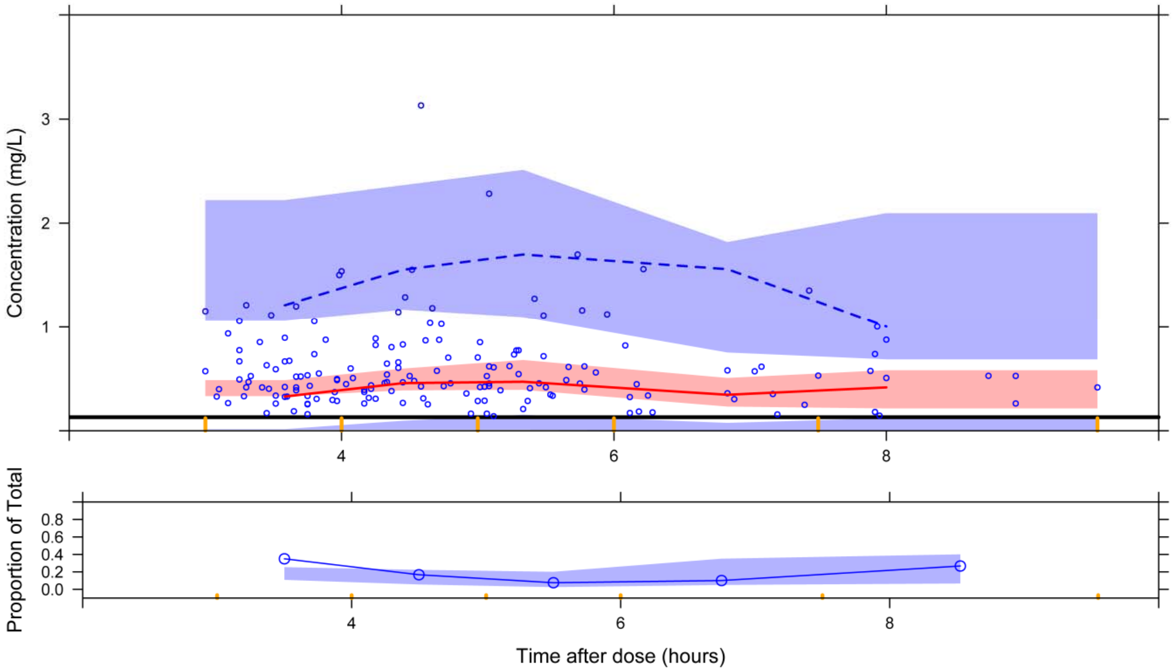

Prediction corrected VPC. In the upper panel the blue rings represent the prediction corrected observed rifampicin concentrations, the lines represent the 2.5th, 50th and 97.5th percentiles of the observed data, and the shaded areas are the 95% confidence intervals of the same percentiles based on data simulated by the final model. In the lower panel the blue rings represent the observed proportions samples below the limit of quantification per bin (indicated by the yellow tick marks), and the shade area represents the 95% confidence intervals of the same proportions based on data simulated by the final model.

Figure E3. Histogram of individual rifampin exposures

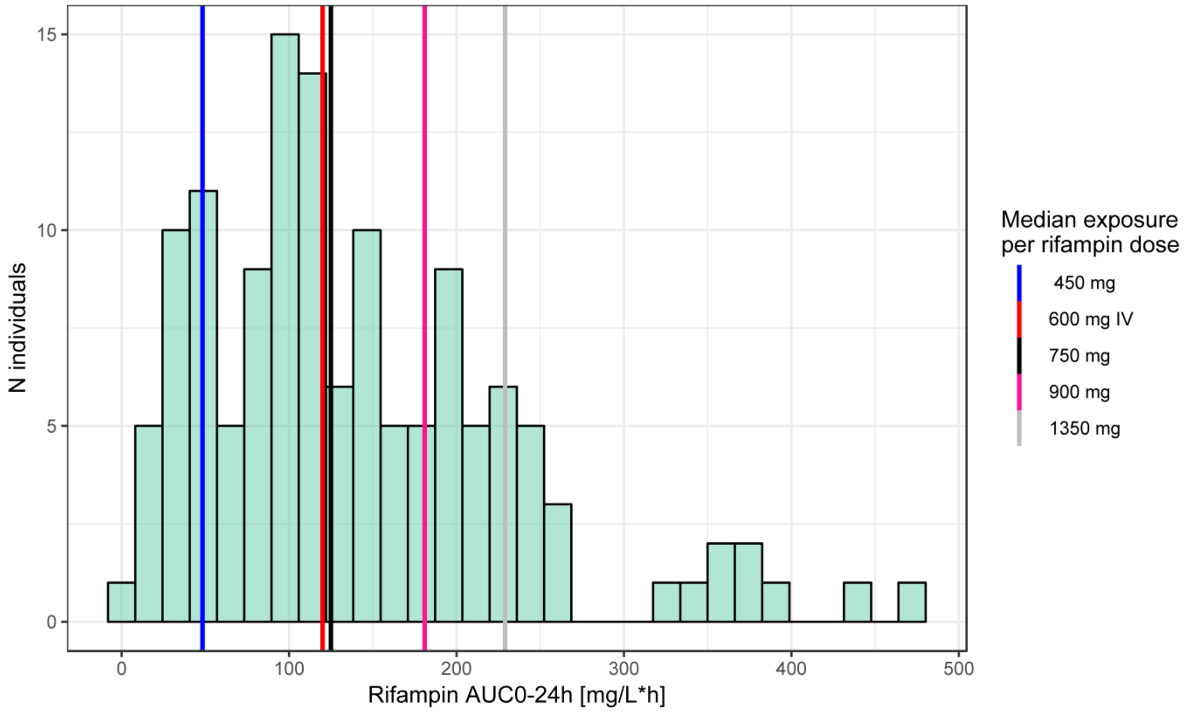

Individual day2±1 rifampin plasma exposures ($AUC_{0\text{-}24h}$) and median exposures per dose group.

Population for dose evaluations

The sex, weight and height distribution in the population used for the dose evaluation was based on the characteristics from a large cohort of Indonesian TBM patients (10), plus an African cohort of pulmonary TB patients (11). The simulation dataset was created by sampling with replacement from the observed patients to create a dataset of 10,000 individuals. Unique individual pharmacokinetic parameters were simulated from the final population pharmacokinetic model, using the same seed number for all evaluated doses.

Survival model

Table E2. Fit of different evaluated base hazard models

Hazard model	Objective function value (OFV)
Constant	774
Weibull	707
Gompertz	714
Exponential decline	683

Table E3. Evaluated relationships between rifampin exposure and base hazard model

Relationship function	N extra estimated parameters	OFV	dOFV*	p-value
None	0	682.5		
AUC power	1	679.5	-3.0	0.08
AUC EMAX	2	678.0	-4.5	0.11
AUC EMAX sigmoid	3	678.0	-4.5	0.21
AUC EMAX fix -1	1	678.0	-4.5	0.03
Cmax EMAX fix -1	1	682.5	0	1
AUCcsf EMAX fix -1	1	681.1	-1.4	0.24

*Difference in OFV compared with the nested base model.

Figure E4. Evaluation of survival model

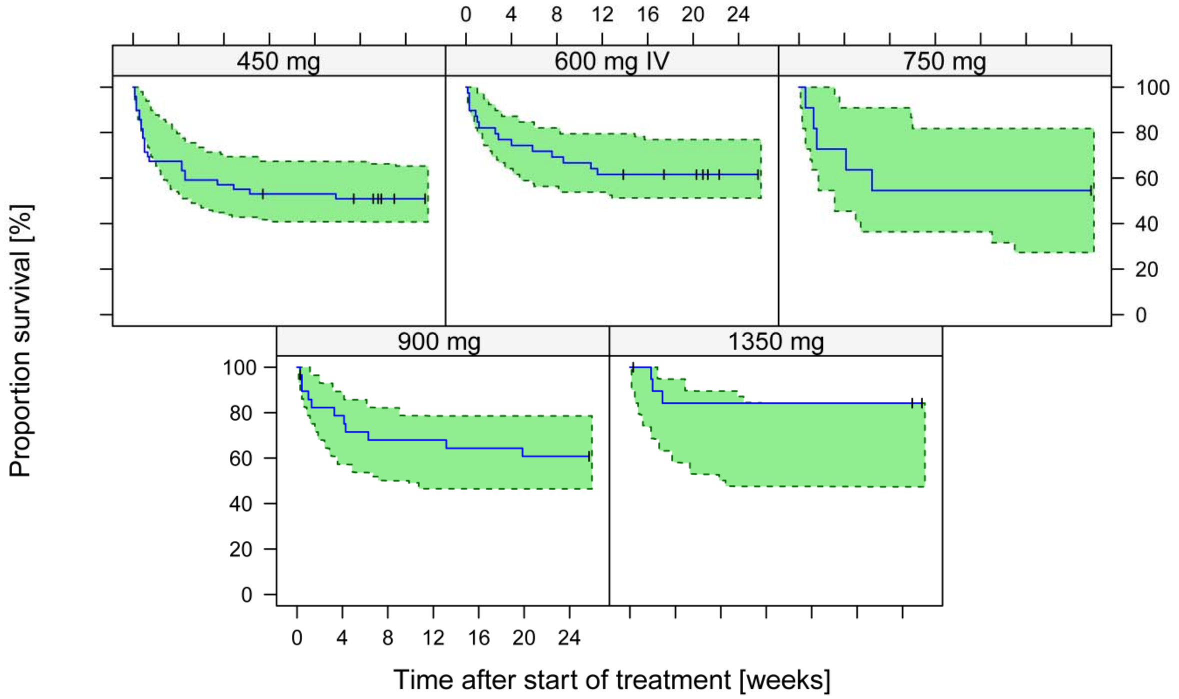

Kaplan-Meier visual predictive check of time-to-event model for survival, stratified on rifampin dose. The solid blue lines are the Kaplan-Meier curves based on the observed data, vertical tick-marks signifies censored data, and the green shaded area outlines the 95% prediction interval based on model simulations.

Albumin concentrations

Albumin concentrations were measured in stored CSF and plasma samples from a cohort of 29 patients with TBM treated at the same clinical site. This cohort partly overlapped with the population included in the trials used for our pharmacokinetics and mortality analysis.

Figure E5. Albumin in CSF and serum

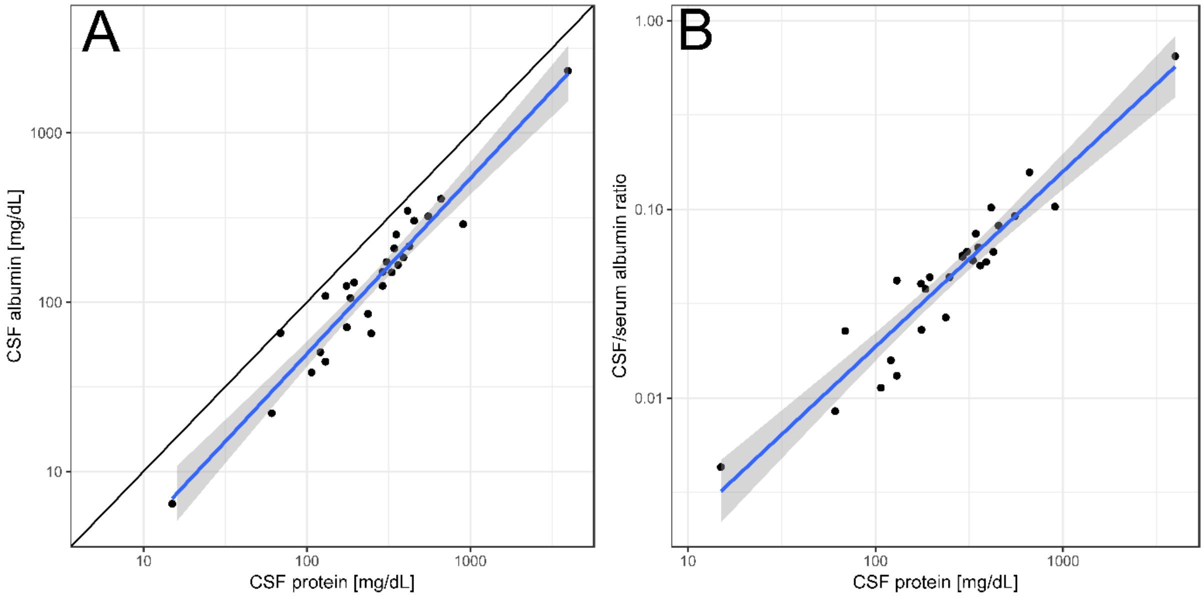

Panel A presents the relation between albumin and total protein concentration in CSF with the line of unity included as a reference and a linear trend-line added ($r^2 = 0.98$). Panel B shows the relation between CSF/serum albumin ratio and total protein concentration in CSF with a linear trend-line added ($r^2 = 0.98$).

NONMEM code pharmacokinetic model

```
;Author: Elin Svensson

$PROBLEM    TBM RIFPK

$INPUT      ID STUDYN RIFDOSE PERIOD DAY TIME TAD DV BLQ EVID AMT FLAG IV RATE CMT
            PREDOS SEX AGE WT HT HIV TBMGRD FFM INITCONC NGT GCS CSFLEUK CSFPROT

$DATA      data.csv IGNORE=@

$SUBROUTINE ADVAN13 TRANS1 TOL=9

$MODEL      NCOMP=5 COMP=(DEPOT,DEFDOSE) COMP=(LIVER) COMP=(CENTRAL,DEFOBS)
COMP=(CSF) COMP=(PERIF)

$PK

NFMV  = FFM
IF(FFM.EQ.-99) NFMV = 44.55
ALLMV = (NFMV/44.55)
ALLMCL = (NFMV/44.55)**0.75

IND = 0
IF(PERIOD.EQ.2) IND = 1

TVCLINTB = THETA(1)*ALLMCL*(1+IND*THETA(8))
TVKM = THETA(2)
TVV = THETA(3)*ALLMV
TVKA = THETA(4)
TVMTT = THETA(5)
TVNN = THETA(6)
TVQ = THETA(10)*ALLMCL
TVVP = THETA(11)*ALLMV

OCC1 = 0
OCC2 = 0
IF(PERIOD.EQ.1) OCC1 = 1
IF(PERIOD.EQ.2) OCC2 = 1

IOVCL = OCC1*ETA(1) + OCC2*ETA(2)
IIVV = ETA(3)
IIVKA = ETA(4)
IOVMTT = OCC1*ETA(5) + OCC2*ETA(6)
IOVF = OCC1*ETA(7) + OCC2*ETA(8)
IIVQ = ETA(9)
IIVPC = ETA(10)
```

```
CLINTB = TVCLINTB
KM = TVKM
V = TVV*(1-(PERIOD-1)*THETA(9))*EXP(IIVV)
Q = TVQ*EXP(IIVQ)
VP = TVVP*(1-(PERIOD-1)*THETA(9))
KA = TVKA*EXP(IIVKA)
IF(IV.EQ.1) KA = 0
MTT = TVMTT*EXP(IOVMTT)
NN = TVNN;*EXP(IIVNN)
KTR = (NN + 1) / MTT
HLCSF = THETA(12)

CSFPROT1 = CSFPROT
IF(CSFPROT1.EQ.-99) CSFPROT1 = 165 ; median period 1, period 2 almost the same
LCSFPROT = LOG10(CSFPROT1)
PC = THETA(14)*(1 + (LCSFPROT-LOG10(165))/LOG10(165)*THETA(15))*EXP(IIVPC)

QH= 50*ALLMCL  ; L/h
FU = 0.2 ; proportion free unbound
VH= 1*ALLMV ; L

; Transit compartment
IF(AMT.GT.0)PD     = AMT          ; PD   = oral DOSE
IF(AMT.GT.0)TDOS   = TIME         ; TDOS = time of DOSE
TAD3               = TIME - TDOS  ; TAD  = time after DOSE
F1 = 0 ; The oral dose via dynamic transit compartment
F3 = 1  ; 100% bioavailability for IV administration
D3 = 1.5 ; Duration of infusion

TVBIO = THETA(7)
PHI = LOG(TVBIO/(1-TVBIO)) + IOVF
BIO= EXP(PHI)/(EXP(PHI) + 1 )

L      = 0.9189385 + (NN + 0.5)*LOG(NN) - NN + LOG(1 + 1/(12*NN))
LBPD  = LOG(BIO*PD)
LKTR  = LOG(KTR)
CUMUL = LBPD + LKTR - L

; Initialize amount in central and peripheral plasma compartments with observed predose
; concentrations
INIT = INITCONC
IF(INIT.EQ.-99) INIT = 0
A_0(1) = 0
A_0(2) = 0
A_0(3) = INIT*V
```

```
A_0(4) = 0
A_0(5) = INIT*VP

$DES

CH = A(2)/VH
CP = A(3)/V

CLINT = CLINTB*KM/(CH + KM)
EH = CLINT*FU/(CLINT*FU + QH)
CLH = EH*QH*EXP(IOVCL)

TEMPO   = T – TDOS
IF(TEMPO.GT.0)THEN
  KTT   = KTR*TEMPO
  DADT(1) = EXP(CUMUL + NN*LOG(KTT) - KTT) - KA*A(1)
ELSE
  KTT   = 0
  DADT(1) = 0
ENDIF

DADT(2) = KA*A(1) - QH*(1-EH)/VH*A(2) + QH/V*A(3)- CLH/VH*A(2)
DADT(3) = QH*(1-EH)/VH*A(2) - QH/V*A(3) - Q/V*A(3) + Q/VP*A(5)
DADT(4) = LOG(2)/HLCSF*(PC*A(3)/V - A(4))
DADT(5) = Q/V*A(3) - Q/VP*A(5)

$ERROR

IPRED1   = A(3)/V
IPRED2   = A(4)

ADD     = SQRT(SIGMA(1,1))   ; ADD error plasma
PROP    = SQRT(SIGMA(2,2))   ; PROP error plasma
ADDCSF  = SQRT(SIGMA(3,3))   ; ADD error CSF

SD1      = SQRT((ADD)**2 + (PROP*IPRED1)**2)
SD2      = SQRT((ADDCSF)**2)

IPRED = IPRED1
SD=SD1
IF(FLAG.EQ.2) THEN
   IPRED = IPRED2
   SD=SD2
ENDIF

IRES   = DV – IPRED
IWRES  = IRES / SD
Y      = IPRED*(1+ EPS(2)) + EPS(1)
```

```
IF(FLAG.EQ.2) Y = IPRED + EPS(3)
IF(ICALL.EQ.4.AND.Y.LE.0) Y = 0.01


$THETA
 (0,40.468) ; 1 CLint L/h
 (0,16.1378,100) ; 2 KM
 (0,7.37836) ; 3 V2 L
 (0,1.40998) ; 4 KA /h
 (0,0.672668) ; 5 MTT h
 (0,4.23047,100) ; 6 NN
 (0,0.77584,1) ; 7 Bioavailability
 (0,0.480062) ; 8 Induction
(0,0.19257,1) ; 9 Difference in V with PERIOD
 (0,93.1391) ; 10 Q, intercompartmental CL
 (0,26.2304) ; 11 Volume periphiral compartment
 (0,2.06319) ; 12 HL equilibrium CSF
 (0,0.0544996,1) ; 13 Penetration coefficent CSF
 (-1,0.630943,10) ; 14 Effect of CSFPROT on PC linear

$OMEGA  BLOCK(1) 0.0597849  ; 1 IOV in CL
$OMEGA  BLOCK(1) SAME
$OMEGA  1.14544  ; 3 IIV in V
$OMEGA  0.575944  ; 4 IIV in KA
$OMEGA  BLOCK(1) 0.379271  ; 5 IOV in MTT
$OMEGA  BLOCK(1) SAME
$OMEGA  BLOCK(1) 1.0329  ; 7 IOV in F
$OMEGA  BLOCK(1) SAME
$OMEGA  0.94784  ; 9 IIV in Q
$OMEGA  0.124736  ; 10 IIV in PC

$SIGMA  0.0100628  ;  ADD ERROR
$SIGMA  0.0572954  ; PROP ERROR
$SIGMA  0.0349855  ; ADD ERROR CSF

$ESTIMATION METHOD=1 INTER MAXEVAL=9999 NSIG=3 SIGL=9 PRINT=1
```

NONMEM code survival model

```
; Author: Elin Svensson

;Sim_start : add/remove for simulation
;$SIZES NO=500 LIM6=500
;Sim_end

$PROBLEM    Joint TBM mortality

$INPUT      ID STUDY DV TIME EVID TYPE SEX AGE WEIGHT HEIGHT HIV TBMG RIFDOSE IV NGTPK1
GCSPK1 NGTPK2 GCSPK2 CSFLEUK CSFPROT CSFBGR GCSB CSFNEUT DROPOUT LASTR CMAX AUC
CMAXCSF AUCCSF

;Sim_start : add/remove for simulation
$DATA      data.csv IGNORE=@ IGNORE(TYPE.EQ.0)
;$DATA     data.csv IGNORE=@ IGNORE(TYPE.EQ.1) IGNORE(DROPOUT.EQ.1)
;Sim_end

$SUBROUTINE ADVAN=6 TOL=9

$MODEL      COMP=(HAZARD)

$PK

RIFAUC = AUC
; Impute AUC for patients with missing PK data
IF(RIFAUC.EQ.-99.AND.RIFDOSE.EQ.450) RIFAUC = 47.61
IF(RIFAUC.EQ.-99.AND.RIFDOSE.EQ.600) RIFAUC = 119.1
IF(RIFAUC.EQ.-99.AND.RIFDOSE.EQ.750) RIFAUC = 123.1
IF(RIFAUC.EQ.-99.AND.RIFDOSE.EQ.900) RIFAUC = 183
IF(RIFAUC.EQ.-99.AND.RIFDOSE.EQ.1350) RIFAUC = 233

BASE = THETA(1)*EXP(ETA(1)) ;the ETA is a placeholder here
COFF = THETA(2)

EC50 = THETA(5)
EFF = 1 - RIFAUC/(RIFAUC + EC50)

$DES

DADT(1)=BASE*EXP(-COFF*(T))*(1+THETA(3)*(GCSB-13))*((AGE/30)**THETA(4))*EFF     ;hazard
```

```
$ERROR

;----------TTE Model------------------------------

CHZ = A(1)        ;cumulative hazard f
SUR = EXP(-CHZ)   ;survival probability
HAZNOW=BASE*EXP(-COFF*(TIME))*(1+THETA(3)*(GCSB-13))*((AGE/30)**THETA(4))*EFF    ;hazard

IF(DV.EQ.0)   Y=SUR         ;censored event (prob of survival)
IF(DV.NE.0)   Y=SUR*HAZNOW  ;prob density function of event



;----------Simulation Model------------------------------

IF(ICALL.EQ.4) THEN

; For new ID
IF(NEWIND.NE.2) THEN
        DV=0
        RTTE = 0
        ORTTE = 0
        CALL RANDOM(2,R)              ; 2nd distribution (uniform)
        USUR=R
ENDIF

; If there was no previous event (SCC or dropout) AND the random
; variable is greater than the proba of survival -> event (SSC)

IF(ORTTE.EQ.0.AND.USUR.GT.SUR) THEN
        DV=1
        RTTE = 1
        ORTTE = 1
ENDIF

; If there was no previous event (SCC or dropout) AND it is the last record -> censoring

IF(ORTTE.EQ.0.AND.LASTR.EQ.1) THEN
        DV=0
        RTTE = 1
        ORTTE = 1
ENDIF

ENDIF
```

```
$THETA  (0,0.0285801) ; 1 Base hazard [per day]
$THETA  (0,0.0333392,5) ; 2 coefficient exponential decline
$THETA  (-0.5,-0.255676,5) ; 3 GCSB effect linear
$THETA  (-1,1.0438,5) ; 4 AGE effect power
$THETA  (0,171.358) ; 5 RIF EC50 effect

$OMEGA  0  FIX

;Sim_start : add/remove for simulation
;$SIMULATION (5988566) (39978 UNIFORM) ONLYSIM NOPREDICTION SUB=100
$ESTIMATION MAXEVAL=9999 METHOD=0 LIKE PRINT=1 SIGL=9 NSIG=3
;Sim_end
```

References

1. Ruslami R, Ganiem AR, Dian S, Apriani L, Achmad TH, van der Ven AJ, et al. Intensified regimen containing rifampicin and moxifloxacin for tuberculous meningitis: an open-label, randomised controlled phase 2 trial. Lancet Infect Dis. 2013;13(1):27-35.
2. Yunivita V, Dian S, Ganiem AR, Hayati E, Hanggono Achmad T, Purnama Dewi A, et al. Pharmacokinetics and safety/tolerability of higher oral and intravenous doses of rifampicin in adult tuberculous meningitis patients. Int J Antimicrob Agents. 2016;48(4):415-21.
3. Dian S, Yunivita V, Ganiem AR, Pramaesya T, Chaidir L, Wahyudi K, et al. Double-Blind, Randomized, Placebo-Controlled Phase II Dose-Finding Study To Evaluate High-Dose Rifampin for Tuberculous Meningitis. Antimicrob Agents Chemother. 2018;62(12).
4. Ruslami R, Nijland HM, Alisjahbana B, Parwati I, van Crevel R, Aarnoutse RE. Pharmacokinetics and tolerability of a higher rifampin dose versus the standard dose in pulmonary tuberculosis patients. Antimicrob Agents Chemother. 2007;51(7):2546-51.
5. Savic RM, Jonker DM, Kerbusch T, Karlsson MO. Implementation of a transit compartment model for describing drug absorption in pharmacokinetic studies. Journal of Pharmacokinetics and Pharmacodynamics. 2007;34(5):711-26.
6. Chirehwa MT, Rustomjee R, Mthiyane T, Onyebujoh P, Smith P, McIlleron H, et al. Model-Based Evaluation of Higher Doses of Rifampin Using a Semimechanistic Model Incorporating Autoinduction and Saturation of Hepatic Extraction. Antimicrob Agents Chemother. 2015;60(1):487-94.
7. Svensson EM, Svensson R, te Brake LH, Boeree M, Heinrich N, Konsten S, et al. The potential for treatment shortening with higher rifampicin doses: relating drug exposure to treatment response in patients with pulmonary tuberculosis. Clin Infect Dis. 2017;Accepted.
8. Svensson RJ, Aarnoutse RE, Diacon AH, Dawson R, Gillespie SH, Boeree MJ, et al. A population pharmacokinetic model incorporating saturable pharmacokinetics and auto-induction for high rifampicin doses. Clin Pharmacol Ther. 2017.
9. Bergstrand M, Hooker AC, Wallin JE, Karlsson MO. Prediction-corrected visual predictive checks for diagnosing nonlinear mixed-effects models. AAPS J. 2011;13(2):143-51.
10. van Laarhoven A, Dian S, Ruesen C, Hayati E, Damen M, Annisa J, et al. Clinical Parameters, Routine Inflammatory Markers, and LTA4H Genotype as Predictors of Mortality Among 608 Patients With Tuberculous Meningitis in Indonesia. J Infect Dis. 2017;215(7):1029-39.
11. Boeree MJ, Heinrich N, Aarnoutse R, Diacon AH, Dawson R, Rehal S, et al. High-dose rifampicin, moxifloxacin, and SQ109 for treating tuberculosis: a multi-arm, multi-stage randomised controlled trial. Lancet Infect Dis. 2017;17(1):39-49.
